# Causal relationship of genetically predicted circulating micronutrients levels with the risk of kidney stone disease: a Mendelian randomization study

**DOI:** 10.3389/fnut.2023.1132597

**Published:** 2023-08-21

**Authors:** Junyi Yang, Weisong Wu, Yirixiatijiang Amier, Xianmiao Li, Wenlong Wan, Xiao Yu

**Affiliations:** Department of Urology, Institute of Urology, Tongji Hospital, Tongji Medical College, Huazhong University of Science and Technology, Wuhan, China

**Keywords:** micronutrient, kidney stone disease, Mendelian randomization, risk factor, genome-wide association study

## Abstract

**Background:**

Current studies have reported conflicting associations between circulating micronutrient levels and kidney stone disease (KSD). We aimed to elucidate the causal relationship between circulating micronutrient levels and KSD by a two-sample Mendelian randomization (MR) analysis.

**Methods:**

Total of 36 single nucleotide polymorphisms (SNPs) from published genome-wide association studies (GWAS) significantly associated with eight micronutrients (vitamin B12, folic acid, magnesium, iron, phosphorus, copper, zinc, and selenium) were used as instrumental variables. The GWAS summary data associated with KSD (8,060 cases and 301,094 controls) were obtained from the FinnGen consortium. Inverse variance weighted was the main MR analysis method. MR-Pleiotropy RESidual Sum and Outlier (MR-PRESSO), weighted median and MR-Egger were used to assess pleiotropy and heterogeneity.

**Results:**

Genetically predicted circulating vitamin B12 and zinc levels were causally associated with the risk of KSD (vitamin B12: OR: 1.17, 95% CI: 1.04–1.32, *p* = 0.008; zinc: OR: 1.15, 95% CI: 1.03–1.28, *p* = 0.015). We found no evidence that other circulating micronutrients were associated with risk of KSD. *p*-value for Cochrane *Q* test, MR Egger intercept test, and MR-PRESSO were >0.05, indicating no significant heterogeneity or horizontal pleiotropy in this MR analysis.

**Conclusion:**

Increasing circulating zinc levels may increase the risk of KSD. More studies are needed to provide evidence on whether genetically predicted circulating vitamin B12 and zinc levels are a risk factor for KSD.

## 1. Introduction

Kidney stone disease (KSD) is a common disease worldwide, and the prevalence is steadily increasing. The prevalence of KSD in the United States population is reported to be about 10% (1). KSD is highly recurrent, with a recurrence rate of approximately 50% within 5–10°years (2). In addition, KSD increases the risk of chronic kidney disease and end-stage kidney disease, which places a significant burden on both patients and the healthcare system (3). However, the risk factors for KSD have not been fully explained. Recent studies have found an association between micronutrients and the development of KSD (4–7). However, due to the limitations of observational studies, the evidence on circulating micronutrients and risk of KSD is susceptible to reverse causality. In fact, most of these studies assessed effects by dietary intake, which may be subject to recall bias and measurement error. Therefore, the causal relationship between circulating micronutrients and the risk of KSD needs a more precise explanation.

Mendelian randomization (MR) is a method for assessing whether there is a potential causal relationship between risk factors and target diseases using genetic variants as instrumental variables (IVs) (8). MR uses random assignment of single nucleotide polymorphisms (SNPs) to simulate randomized trials in a population and is independent of environmental and other unknown confounding factors, thus overcoming potential confounding and reverse causality (9). Two MR studies have investigated the casual relationship between circulating calcium, retinol, beta-carotene, α-tocopherol, lycopene, and vitamins B6, C, D with the risk of KSD (10, 11). In this study, we used a two-sample MR analysis to assess the causal relationship between some other circulating micronutrients and the risk of KSD.

## 2. Materials and methods

### 2.1. Study design

**FIGURE 1:**
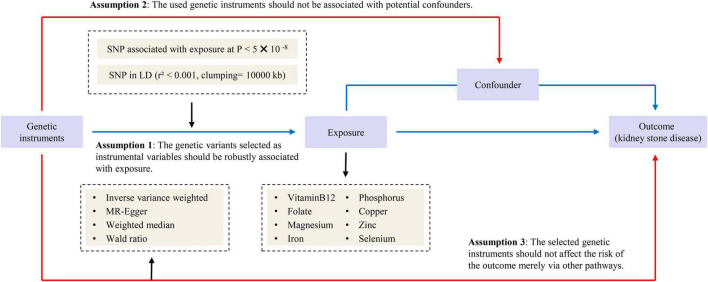
Overview and assumptions of the Mendelian randomization study design.

Our study followed the strengthening the reporting of observational studies in epidemiology – mendelian randomization (STROBE-MR) statement used to report Mendelian randomization (MR) research (12). Previously collected and published data were applied to this study and analyzed. Therefore, no additional ethical approval was needed. An overview of the study design is presented in Figure 1.

### 2.2. Genetic instrument selection

We searched the PubMed and the genome-wide association studies (GWAS) catalog^1^ for published GWAS on circulating levels of various micronutrients and obtained summary statistics for eight micronutrients: vitamin B12 (13), folate (13), phosphorus (14), magnesium (15), iron (16), copper (17), selenium (17), and zinc (17). Then we screened SNPs that were significantly associated with circulating micronutrient levels and were not in a chain imbalance by a genome-wide significance threshold (*p*-value < 5E-08). All SNPs were clumped based on linkage disequilibrium (LD), defined by *r*^2^ < 0.001 with clumping window > 10,000°kb. The detailed information of GWAS related to eight exposures are listed in Supplementary Table 1. In addition, we searched the PhenoScanner database^2^ to exclude SNPs that might be associated with confounding factors (threshold of *p*-value = 5E-08, *r*^2^ = 0.8) (18). The confounding factors we observed that might be associated with outcome included sodium in urine (19), glomerular filtration rate creatinine (20), serum urate (21), glycated hemoglobin (22), and serum calcium (10) (Supplementary Table 2). We searched SNiPA^3^ to find the proxy-SNP (*r*^2^ > 0.8) when SNPs couldn’t be found in the outcome dataset.

### 2.3. Data source for kidney stone disease

Genome-wide association studies summary data on kidney stone disease (KSD) were obtained from the FinnGen consortium.^4^ 8,060 cases (defined by N20-N23 in ICD10) and 301,094 controls were used in the seventh release of the FinnGen consortium, which removed individuals with unclear gender, high genotype deletion rates (> 5%), excess heterozygosity (±4 SD) and non-Finnish ancestry.

### 2.4. Statistical analysis

After harmonizing single nucleotide polymorphisms (SNPs) with the same allele, we employed inverse variance weighted (IVW), MR-Egger and weighted median to perform two-sample MR analysis. We applied the random-effects IVW method as the main statistical model, which is similar to a meta-analysis of single SNP-specific Wald ratio (23). MR-Egger and weighted median method are used as secondary statistical models only when the number of SNPs is greater than three. MR-Egger tolerates potential pleiotropy and provides conservative estimates of causal effects (24). Weighted median allows for 50% invalid IVs and provides reliable estimates of causal effects (25). When only one SNP is available, the Wald ratio method is used to deduce the effect of a single IV on KSD. Considering the possible sample overlap between exposure and outcome data, we calculated the F-statistic to measure the strength of the IVs (26). Cochrane’s *Q* test was used to assess heterogeneity and *p*-value < 0.05 indicated heterogeneity. MR-Egger intercept was used to assess pleiotropy and *p*-value < 0.05 indicated pleiotropic bias. The leave-one-out analysis and the MR-Pleiotropy Residual Sum and Outlier method (MR-PRESSO) were used to assess whether the results were influenced by outlier SNPs. In addition, forest plots of MR analyses were also provided. All analyses were performed using the packages TwoSampleMR (version 0.5.6) and MR-PRESSO (version 1.0) in R (version 4.2.1).

## 3. Results

### 3.1. Mendelian randomization estimates

**FIGURE 2:**
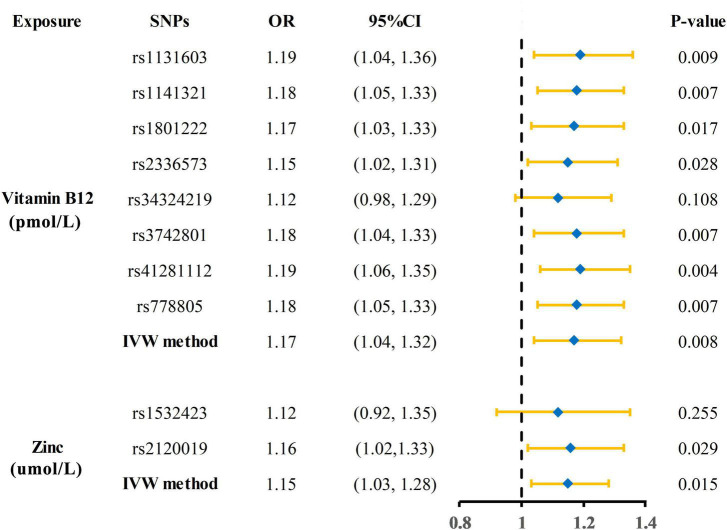
Forest plot showing results of each SNPs from Mendelian randomization analyses of vitamin B12 and zinc and risk of kidney stone disease. SNPs, single nucleotide polymorphisms; OR, odds ratio; CI, confidence interval; IVW, inverse variance weighted.

**FIGURE 3:**
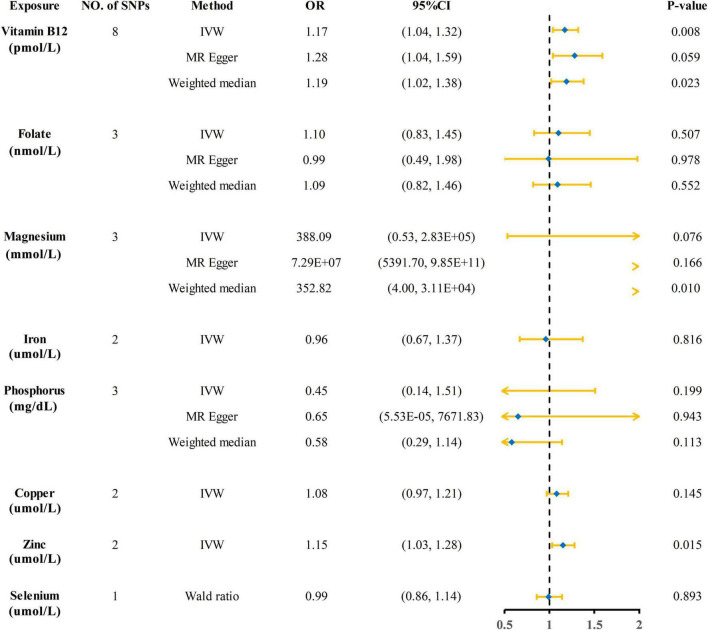
Forest plot showing results from Mendelian randomization analyses of eight circulating micronutrient concentrations and risk of kidney stone disease. SNPs, single nucleotide polymorphisms; OR, odds ratio; CI, confidence interval; IVW, inverse variance weighted.

The Mendelian randomization (MR) estimates obtained by the inverse variance weighted (IVW) method suggested that predicted circulating micronutrient concentrations of Vitamin B12 (OR: 1.17, 95% CI: 1.04–1.32, *p* = 0.008) and zinc (OR: 1.15, 95% CI: 1.03–1.28, *p* = 0.015) were suggestively associated with a high risk of KSD (Figure 2). We found no evidence that circulating micronutrient concentrations of folate (OR: 1.10, *p* = 0.507), magnesium (OR: 388.09, *p* = 0.076), iron (OR: 0.96, *p* = 0.816), phosphorus (OR: 0.45, *p* = 0.199), copper (OR: 1.08, *p* = 0.145), and selenium (OR: 0.99, *p* = 0.893) are associated with the risk of KSD (Figure 3 and Supplementary Table 3).

### 3.2. Sensitive analysis

As shown in Supplementary Table 4, the total F statistics of SNPs for vitamin B12 and zinc are 219.21 and 28.78, respectively. Cochrane’s *Q* test and MR-Egger intercept showed no heterogeneity or horizontal pleiotropy in analyses of vitamin B12 and zinc (both *p*-value > 0.05). In MR-PRESSO analysis, no single SNP showed an abnormal effect on kidney stone disease (*p*-value = 0.791). Scatter plot, funnel plot, and leave-one-out plot showed that the results from Mendelian randomization analyses of vitamin B12 and risk of KSD was robust under different sensitivity analyses (Supplementary Figure 1).

## 4. Discussion

Kidney stone disease is a disease with a high incidence and recurrence rate. To explore possible preventive measures furtherly, we performed a two-sample MR analysis of large GWAS summary data on eight circulating micronutrients levels. Finally, we found that genetically predicted circulating vitamin B12 and zinc may be potential risk factors for KSD.

Vitamin B12, also known as cobalamin, is often involved in biochemical reactions as a cofactor and the production of red blood cells (27). Previous studies have shown that it may be associated with hematological disorders and neurological disorders and cardiovascular disease (28, 29). There are few clinical studies on vitamin B12 and KSD. A single-center retrospective study found more oxalate crystals in renal tubular epithelium cells (RTECs) in burn patients treated with hydroxocobalamin (30). Hydroxocobalamin is an active form of vitamin B12. If hydroxocobalamin promotes the production of oxalate crystals in RTECs, it may support our analysis that circulating vitamin B12 levels are a potential risk factor for KSD. However, most current studies are more supportive of a protective role of circulating vitamin B12 in the kidney. Homocysteine is a non-essential, sulfur-containing amino acid that is involved in the metabolism of methionine. Normally, approximately 50% of homocysteine is remethylated to methionine via the folate/B12 pathway (31). Vitamin B12 deficiency can lead to hyperhomocysteinemia, promoting kidney injury by inducing oxidative stress and inflammatory responses. Therefore, vitamin B12 may inhibit kidney stone formation by promoting homocysteine metabolism and reducing oxidative stress and inflammatory damage, which contradicts the result of our analysis. In addition, although our study excluded SNPs associated with confounding factors, the association between vitamin B12 and KSD may be confounded by the consumption of foods of animal origin. The European Association of Urology guidelines consider high animal protein intake to be an important risk factor for KSD. But vitamin B12 is mainly concentrated in animal tissues, and humans need to consume foods of animal origin to obtain vitamin B12. The amount and type of protein also determines the degree of vitamin B12 absorption (32). We speculated that this contradictory result may be due to the release of vitamin B12 from RTECs injury. Before the formation of kidney stone, RTECs are often already damaged under the influence of multiple factors. The liver and kidney are the main storage organs for vitamin B12, which is released when RTECs are damaged, resulting in an increase in circulating vitamin B12 levels. Therefore, the causal relationship between circulating vitamin B12 levels and KSD may be confounded by the causal relationship between RTECs injury and KSD. In conclusion, with the available evidence, we cannot directly determine that genetically predicted circulating vitamin B12 levels are a potential risk factor for kidney stone formation.

Zinc is an essential trace metal required for biological growth and is involved in the regulation of immunomodulatory functions (33). Previous findings on the association between zinc and KSD were contradictory. A randomized controlled trial enrolling 3,640 subjects showed that men who received high doses of zinc supplementation had a higher risk of KSD compared to the placebo group (34). This is similar to the result of our study. A cross-sectional study enrolling 15,444 subjects showed that higher zinc intake was associated with an increased risk of KSD (35). In contrast, cross-sectional data from adult participants in the NHANES showed that dietary zinc intake and serum zinc levels were negatively associated with the prevalence of KSD (6). Another case-control study including 30 adolescents found that reduced dietary zinc intake was independently associated with the occurrence of KSD (36). However, a prospective study of three cohorts did not report any association between zinc intake and risk of KSD (37). A recent systematic review similarly reported no significant effect of zinc intake on the risk of stone formation (5). The inconsistency of the above results may be due to some limitations. First, these studies used dietary zinc levels rather than circulating zinc levels as an indicator. The results may be affected by the absorption levels of different subject populations. Second, these studies were mainly observational studies and the results may be affected by measurement error, subject recall bias, potential confounders and reverse causality. Third, different zinc concentrations may have different effects on crystal formation. Low concentrations of zinc can inhibit the growth of calcium phosphate, while at higher concentrations zinc can promote its formation (38). The exact mechanism of zinc in KSD is still uncertain, but several studies have identified a potential role for zinc in kidney stone formation. Carpentier et al. (39) found a dramatic increase in zinc in Randall’s plaque, suggesting that zinc could promote interstitial calcium phosphate deposition. Ozgurtas et al. (40) suggested that urinary zinc increased excretion may act as a trigger for crystal formation, which may then cause urolithiasis. Bazin et al. (41) found that zinc and strontium accounted for 91% of the heavy metal composition of kidney stones, and zinc can replace calcium in crystals because of similarity in charge and size. Chi et al. (42) repressed the zinc transporter protein gene and inhibited stone formation in a Drosophila melanogaster model for ectopic calcification, suggesting that zinc may play a key role in driving heterogeneous nucleation.

This study has several strengths. First, we added to the causal relationship between circulating micronutrients and Kidney stone disease (KSD) and reported for the first time that circulating vitamin B12 and zinc levels are potential risk factors for KSD. Second, this study is a two-sample MR analysis and excludes SNPs that may be associated with outcomes, overcoming the limitations of observational studies, including confounders, reverse causality, and recall bias. Third, the bias caused by sample overlap in this study was small. Even though the vitamin B12 and zinc exposed populations overlapped completely with the outcome population, the overlap rates were 14.7 and 0.8%, respectively. According to Burgess’ simulations, the Type I errors were both less than 0.05 (26).

Our study has some limitations. First, we did not adjust for animal protein intake as a potential confounder, which may have caused pleiotropy bias. Second, the sample was limited to European populations to reduce the possible effect of stratified populations. Third, the statistical power was less than 80% due to the small sample size. Finally, due to the paucity of currently available GWAS data on circulating micronutrient levels, our findings are based on a small number of IVs. More comprehensive GWAS data are needed to refine this study in the future.

## 5. Conclusion

This study provides genetic evidence for a causal relationship between circulating micronutrient concentrations and KSD risk. Increasing circulating zinc levels may increase the risk of KSD. More studies are needed to provide evidence on whether genetically predicted circulating vitamin B12 and zinc levels are a risk factor for KSD.

## Data availability statement

The original contributions presented in this study are included in the article/Supplementary material, further inquiries can be directed to the corresponding author.

## Author contributions

JY prepared and drafted the manuscript. XY obtained the funding for the study and provided the critical revision of the manuscript for the important intellectual content. WSW and YA assisted in obtaining data for the review article and revised the manuscript. XL and WLW confirmed the authenticity of all the raw data. All authors read and approved the final manuscript.

## Footnotes

1
www.ebi.ac.uk/gwas

2
www.phenoscanner.medschl.cam.ac.uk

3
http://snipa.helmholtz-muenchen.de/snipa3/

4
www.finngen.fi/en
