# Supplementary material for: Causal relationship of genetically predicted circulating micronutrients levels with the risk of kidney stone disease: a Mendelian randomization study
Source: Front Nutr. 2023 Aug 21;10:1132597. doi: 10.3389/fnut.2023.1132597 (PMC10476526; doi:10.3389/fnut.2023.1132597)

Supplementary Information

**Causal relationship of genetically predicted circulating micronutrients levels with the risk of kidney stone disease: A Mendelian Randomization Study**

Junyi Yang ^a^, Weisong Wu ^a^, Yirixiatijiang Amier ^a^, Xianmiao Li ^a^, Wenlong Wan ^a^, Xiao Yu ^a,^*

a Department of Urology, Institute of Urology, Tongji Hospital, Tongji Medical College, Huazhong University of Science and Technology, Wuhan 430030, China

**Supplementary Table S1** Summary information for 36 SNPs of 8 nutrient concentrations.

| Exposure | SNPs | EA | OA | EAF | Beta | SE | P-value | Sample size^#^ |
| --- | --- | --- | --- | --- | --- | --- | --- | --- |
| Vitamin B12 | rs1131603 | C | T | 0.06 | 0.222 | 0.015 | 2.11E-48 | 45576 |
|  | rs1141321 | C | T | 0.63 | 0.070 | 0.007 | 5.11E-25 | 45576 |
|  | rs1801222 | G | A | 0.59 | 0.119 | 0.007 | 7.24E-74 | 45576 |
|  | *rs2270655 | G | C | 0.94 | 0.099 | 0.015 | 5.68E-12 | 45576 |
|  | rs2336573 | T | C | 0.03 | 0.313 | 0.019 | 2.89E-60 | 45576 |
|  | rs34324219 | C | A | 0.88 | 0.235 | 0.011 | 2.54E-109 | 45576 |
|  | rs3742801 | T | C | 0.29 | 0.053 | 0.007 | 2.28E-13 | 45576 |
|  | rs41281112 | C | T | 0.95 | 0.181 | 0.015 | 4.6E-34 | 45576 |
|  | rs602662 | A | G | 0.60 | 0.171 | 0.007 | 8.15E-138 | 45576 |
|  | rs778805 | A | G | 0.25 | 0.050 | 0.008 | 1.04E-10 | 45576 |
| Folate | rs1801133 | G | A | 0.67 | 0.114 | 0.008 | 6.65E-53 | 37341 |
|  | rs652197 | C | T | 0.18 | 0.069 | 0.010 | 5.73E-13 | 37341 |
|  | rs76630415 | G | T | 0.21 | -0.037 | 0.007 | 2.40E-08 | 64979 |
| Magnesium | rs11144134 | C | T | 0.08 | 0.011 | 0.001 | 8.21E-15 | 23829 |
|  | rs13146355 | A | G | 0.44 | 0.005 | 0.001 | 6.27E-13 | 23829 |
|  | rs3925584 | T | C | 0.55 | 0.006 | 0.001 | 5.2E-16 | 23829 |
|  | rs4072037 | T | C | 0.54 | 0.010 | 0.001 | 2.01E-36 | 23829 |
|  | rs448378 | A | G | 0.53 | 0.004 | 0.001 | 1.25E-08 | 23829 |
|  | ^✝^rs7965584 | A | G | 0.71 | 0.007 | 0.001 | 1.05E-16 | 23829 |
| Iron | *rs1799945 | C | G | 0.85 | -0.189 | 0.010 | 1.1E-81 | 48972 |
|  | rs1800562 | A | G | 0.07 | 0.328 | 0.016 | 2.72E-97 | 48972 |
|  | rs7385804 | A | C | 0.62 | 0.064 | 0.007 | 1.36E-18 | 48972 |
|  | rs8177240 | T | G | 0.67 | -0.066 | 0.007 | 6.65E-20 | 48972 |
|  | rs855791 | A | G | 0.45 | -0.181 | 0.007 | 1.32E-139 | 48972 |
| Phosphorus | rs1697421 | A | G | 0.49 | 0.050 | 0.005 | 1.14E-27 | 16264 |
|  | rs17265703 | A | G | 0.85 | 0.036 | 0.006 | 4.32E-09 | 16264 |
|  | *rs2970818 | A | T | 0.09 | 0.047 | 0.008 | 4.38E-09 | 16264 |
|  | rs9469578 | C | T | 0.92 | 0.059 | 0.009 | 1.11E-11 | 16264 |
|  | rs947583 | C | T | 0.29 | 0.035 | 0.005 | 3.45E-12 | 16264 |
| Copper | rs1175550 | A | G | 0.78 | -0.198 | 0.032 | 5.03E-10 | 2603 |
|  | rs2769264 | G | T | 0.16 | 0.313 | 0.034 | 2.63E-20 | 2603 |
| Zinc | rs1532423 | A | G | 0.37 | 0.178 | 0.026 | 6.40E-12 | 2603 |
| (2603) | rs2120019 | T | C | 0.79 | 0.287 | 0.033 | 1.55E-18 | 2603 |
|  | ^※^rs4826508 | T | C | 0.27 | 0.210 | 0.030 | 1.40E-12 | 2603 |
| Selenium | rs921943 | T | C | 0.29 | 0.246 | 0.023 | 9.40E-28 | 2603 |
|  | ^※^rs7700970 | T | C | 0.30 | 0.212 | 0.024 | 1.72E-18 | 2603 |

^#^The sample was limited to European populations.

*These SNPs were excluded for being palindromic with intermediate allele frequencies.

^✝^rs7965584 was not available in the outcome dataset and rs10858939 was found to replace them, respectively.

^※^rs4826508 and rs7700970 was excluded for linkage disequilibrium.

Abbreviations: SNPs: single nucleotide polymorphisms; OA: other allele; EA: effect allele; EAF: effect allele frequency; SE: standard error.

**Supplementary Table S2** Characteristics of 7 SNPs excluded for strong association with confounders

| SNPs | EA | OA | EAF | Beta | SE | P-value | Confounders |
| --- | --- | --- | --- | --- | --- | --- | --- |
| rs602662 | A | G | 0.60 | -0.014 | 0.002 | 2.12E-09 | Sodium in urine |
| rs13146355 | A | G | 0.44 | 0.012 | 0.001 | 3.00E-37 | Glomerular filtration rate creatinine |
| rs3925584 | T | C | 0.55 | -0.008 | 0.001 | 7.60E-18 | log eGFR creatinine |
| rs4072037 | T | C | 0.54 | -0.035 | 0.006 | 3.91E-09 | Serum urate |
| rs1800562 | A | G | 0.07 | NA | NA | 3.00E-20 | Hemoglobin a glycosylated |
| rs855791 | A | G | 0.45 | NA | NA | 3.00E-14 | Hemoglobin a glycosylated |
| rs17265703 | A | G | 0.85 | NA | NA | 1.80E-18 | Serum calcium |

Abbreviations: SNPs: single nucleotide polymorphisms; OA: other allele; EA: effect allele; EAF: effect allele frequency; SE: standard error.

**Supplementary Table S3** Summary information for 24 SNPs of 8 nutrient concentrations and risk of kidney stone disease.

| Exposure | SNPs | EA | OA | OR | 95%CI | P-value | | |
| --- | --- | --- | --- | --- | --- | --- | --- | --- |
| Vitamin B12 | rs1131603 | C | T | 1.19 | (1.04, 1.36) | | 0.009 |  |
|  | rs1141321 | C | T | 1.18 | (1.05, 1.33) | | 0.007 |  |
|  | rs1801222 | G | A | 1.17 | (1.03, 1.33) | | 0.017 |  |
|  | rs2336573 | T | C | 1.15 | (1.02, 1.31) | | 0.028 |  |
|  | rs34324219 | C | A | 1.12 | (0.98, 1.29) | | 0.108 |  |
|  | rs3742801 | T | C | 1.18 | (1.04, 1.33) | | 0.007 |  |
|  | rs41281112 | C | T | 1.19 | (1.06, 1.35) | | 0.004 |  |
|  | rs778805 | A | G | 1.18 | (1.05, 1.33) | | 0.007 |  |
| Folate | rs1801133 | G | A | 1.17 | (0.70, 1.94) | | 0.547 |  |
|  | rs652197 | C | T | 1.08 | (0.79, 1.48) | | 0.610 |  |
|  | rs76630415 | G | T | 1.09 | (0.82, 1.45) | | 0.566 |  |
| Magnesium | rs11144134 | C | T | 1.29E+05 | (415.50, 4.01E+07) | | 5.83E-05 |  |
|  | rs448378 | A | G | 0.18 | (5.97E-05, 526.71) | | 0.672 |  |
|  | rs10858939 | A | C | 106.61 | (0.88,1.30E+04) | | 0.057 |  |
| Iron | rs7385804 | A | C | 1.12 | (0.68, 1.84) | | 0.652 |  |
|  | rs8177240 | T | G | 0.81 | (0.48, 1.36) | | 0.420 |  |
| Phosphorus | rs1697421 | A | G | 1.15 | (0.57, 2.30) | | 0.691 |  |
|  | rs9469578 | C | T | 0.28 | (0.09, 0.85) | | 0.025 |  |
|  | rs947583 | C | T | 0.41 | (0.07, 2.41) | | 0.323 |  |
| Copper | rs1175550 | A | G | 1.15 | (0.96, 1.37) | | 0.130 |  |
|  | rs2769264 | G | T | 1.05 | (0.92, 1.20) | | 0.494 |  |
| Zinc | rs1532423 | A | G | 1.12 | (0.92, 1.35) | | 0.255 |  |
|  | rs2120019 | T | C | 1.16 | (1.02,1.33) | | 0.029 |  |
| Selenium | rs921943 | T | C | 0.99 | (0.86, 1.14) | | 0.893 |  |

Abbreviations: SNPs: single nucleotide polymorphisms; OA: other allele; EA: effect allele; OR: odds ratio; CI: confidence interval.

**Supplementary Table S4** F-statistic, Cochrane’s Q test, MR-Egger intercept and MR-PRESSO Mendelian randomization analyses of Vitamin B12 and Zinc.

| Exposure | Sample size | R^2^ | F | Cochrane’s Q test^1^ (P-value) | MR-Egger intercept^2^ (P-value) | MR-PRESSO^3^  (P-value) |
| --- | --- | --- | --- | --- | --- | --- |
| Vitamin B12 | 45576 | 3.71% | 219.21 | 0.812 | 0.347 | 0.791 |
| Zinc | 2603 | 3.22% | 28.78 | 0.725 | NA | NA |

^1^The Cochrane’s Q test was used to test heterogeneity.

^2^The MR-Egger intercept was used to test horizontal pleiotropy.

^3^The MR-PRESSO method was used to detect the existence of outlier IVs.

F = R^2^(N-K-1)/[K(1–R^2^)], R^2^ = 2 × (1–EAF) × EAF × β^2^, where EAF is the effect allele frequency, β is the estimated effect on exposures, N is the sample size of the GWAS and K is the number of SNPs.

Abbreviations: MR-PRESSO: the Mendelian Randomization Pleiotropy RESidual Sum and Outlier.

**Supplementary Figure S1** MR-Egger estimates of significant results from vitamin B12 on kidney stone disease. (a) Scatter plot from genetically predicted vitamin B12 on kidney stone disease. (b) Funnel plot from genetically predicted vitamin B12 on kidney stone disease. (c) Leave-one-out plot from genetically predicted vitamin B12 on kidney stone disease.


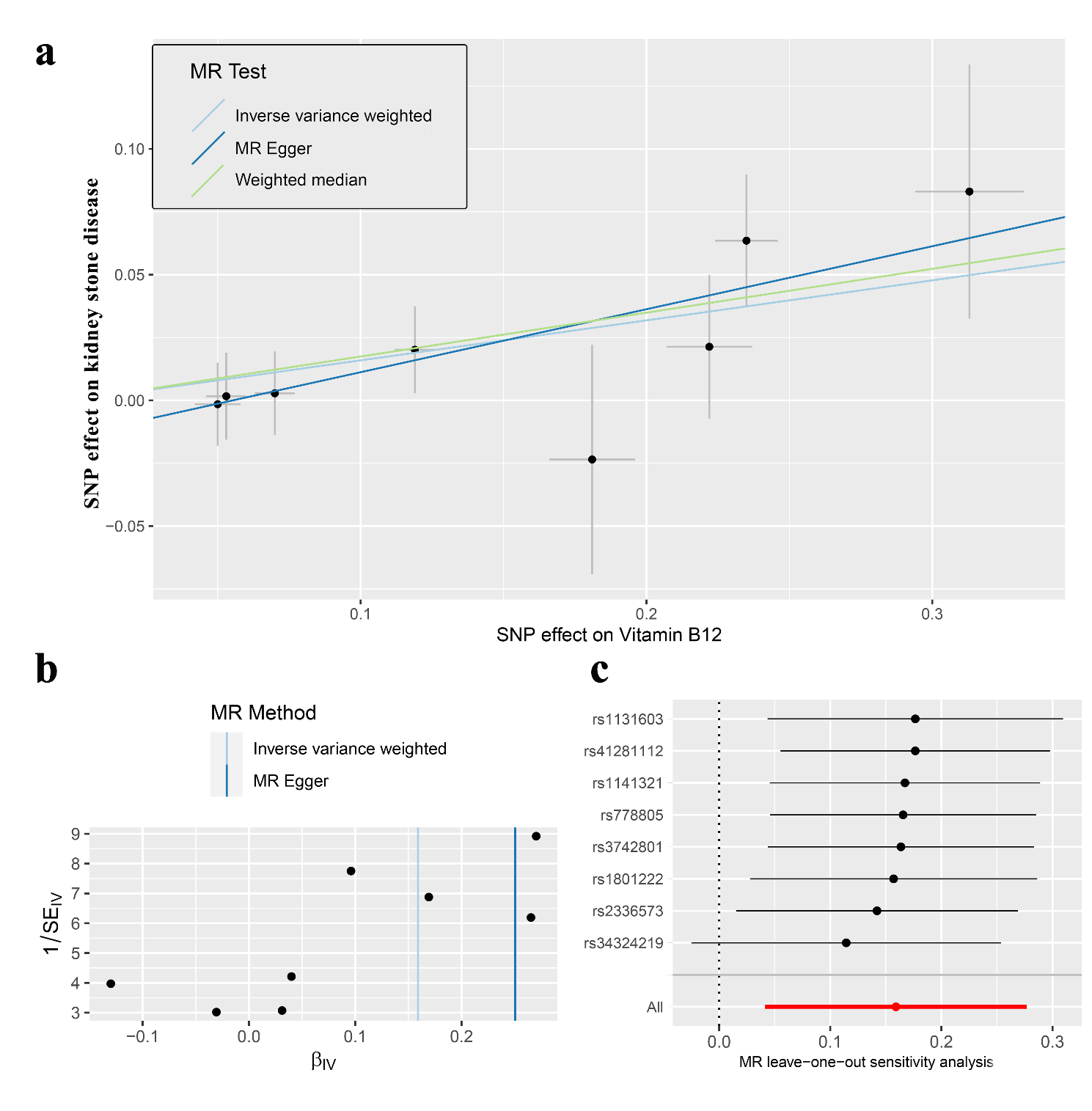

Supplement: Supplementary file 1 [file Data_Sheet_1.docx]
